# Insights into the use of telemedicine in primary care in times of the SARS-CoV-2 pandemic - a cross-sectional analysis based on the international PRICOV-19 study in Austria

**DOI:** 10.1186/s12875-023-02113-6

**Published:** 2023-10-24

**Authors:** Florian Odilo Stummer, Lisa Voggenberger, Maria de la Cruz Gomez Pellin, Esther van Poel, Sara Willems, Kathryn Hoffmann

**Affiliations:** 1 Department of Primary Care Medicine, Center for Public Health, Medical University of Vienna, Vienna, Austria; 2 Institute for General Medicine, Johannes-Kepler-University, Linz, Austria; 3 Department of Public Health and Primary Care, Ghent University, Ghent, Belgium

**Keywords:** Primary health care, General practice, Video consultations, Telemedicine, SARS-CoV-2 pandemic, COVID-19, Austria, PRICOV-19

## Abstract

**Background:**

The SARS-CoV2 pandemic as well as the implementation of public health measures to decrease the spread of the virus re-sparked the call for “virtual” health or “distance” treatments. This paper aimed to assess the use of video consultations, the up-to-dateness of practice websites, and the views of GPs on whether eHealth is a positive aspect for the future of their practices in publicly -funded primary healthcare facilities in Austria.

**Methods:**

The cross-sectional online questionnaire, part of the PRICOV-19 study, was conducted from December 2020 until July 2021. We randomly recruited 176 GP practices across Austria. Descriptive statistics as well as binary logistic regression models were applied to examine the associations between telemedicine use and practice factors.

**Results:**

Compared with before the pandemic (3.8%), 7.6% of publicly funded GP practices have been using video consultations since the pandemic. In line with this, 93.9% of the practices had no increase in video consultation use. Fewer than half (44.3%) had an up-to-date webpage, and 27.8% assumed that the pandemic might have been a positive driver for eHealth in their practices. Positive associations with video consultation use could be found in practices with fewer patients aged 70 years and over than the average and more patients with chronic diseases than the average.

**Conclusion:**

The use of video consultations in general practice and the readiness for other telemedicine approaches are both very low in Austria. Austria has to urgently follow the example of countries with a transparent and comprehensive national digital health strategy that includes video consultation. Without a proper payment system, patient inclusion, and support with regard to administrative and organizational aspects, no substantial change will occur in spite of an increase in need due to the pandemic and changes in the patient population.

## Background

### Telemedicine in primary care during the SARS-CoV-2 pandemic

Since January 2020, the severe acute respiratory syndrome coronavirus 2 (SARS-CoV-2) pandemic has had the world within its grasp. The pressure on health-care systems worldwide has resulted in major challenges and the fragmentation of provision and support processes [1].

Fast policy response has been vital, as threats throughout the systems have demanded sustainable solutions that guarantee planning security [2]. As primary care is one of the main pillars for maintaining health care for a wide population range in a pandemic situation, the use of telemedicine is a vital support component to keep healthcare provision sustainable.

The use of digital technologies to improve sustainable care routines, however, have in many countries not yet adapted to the needs or the capacity of patient populations because of widespread skepticism regarding telemedicine [3]. However, telephone and video consultation (VC) have already been rolled out in several countries as part of their national digital health strategies [4].

The implementation of public health measures to decrease the spread of the virus, such as quarantine, isolation, and physical distancing, once again sparked the call for “virtual” health or “distance” treatments. For example, teleconsultations were remembered as having provided good support in previous pandemics, such as those of Ebola or SARS [5, 6].

Thus, for several countries, it became during the SARS-CoV-2 pandemic an additional strategy for serving primary healthcare demand [4]. Where it existed, this technology contributed to the quality of health-care services in primary health care and strengthened the monitoring, surveillance, and detection of new COVID-19 cases as well as the maintenance of health care for persons with other diseases. It was also demonstrated to contribute to reducing patients’ anxiety due to isolation and maintain contact between health professionals and patients with SARS-CoV-2, thereby allowing timely attention to the most urgent cases and those with chronic diseases [7].

However, organizational case studies have shown that VC can disrupt, for example, the routines and processes of GP practices, leading to additional consultation times. Moreover, many physicians have expressed concerns about technical as well as privacy, safety, and accountability issues that hinder the use of telemedicine [8–11].

Additionally, VC needs sustainable implementation solutions, but weak broadband connection, lack of or outdated equipment, and lack of training for caregivers and patients make it impossible to guarantee comprehensive and permanent implementation, leading to an increase in overall costs to the health-care system and to GPs [10–12]. Another issue are the general conditions surrounding technical frameworks, the data protection framework, administrative data protection, administrative and organizational frameworks and conditions, the applicability for both patients and physicians, quality criteria and remuneration [13].

Moreover, an important issue to consider when it comes to using telemedicine concerns financing. Whether the widespread implementation of telemedicine will create an economic burden or an economic opportunity for health-care providers is as yet still under debate. While VC has the potential to improve efficiency and, after an initial phase, save time and therefore money for physicians, studies that have investigated the economic consequences of telemedicine suffer from poor quality and considerable heterogeneity, making comparisons or meta-analyses of their data impossible. This was the conclusion of a systematic review published in 2017 [14].

### The Austrian situation

In Austria, efforts to promote telemedicine applications have been underway for some time in both the public and private sectors, and the SARS-CoV-2 pandemic resulted in an unexpected upswing and new developments in this field.

Nevertheless, until recently, payment for Austrian physicians accommodating their patients by offering VC was not guaranteed. This means that the new tariff plan was introduced nine months after COVID-19 breakout and included a reimbursement for the use of VC equivalent to face-to-face appointments. However, those GPs who already provided VC were not paid for these nine months [15].

This is important because Austria is a Bismarck-system country without gatekeeping or a list-system. GPs are generally self-employed and remunerated by a mixed payment model that is predominantly fee for service [16]. The first step in this direction was undertaken in the year 2021 by the National Health Insurance of Austria (Österreichische Gesundheitskasse), which changed its policy and implemented the equivalency of VC with regular in-person office visits with regard to payment for the physician. But only if the related patients was already seen before face-to-face [15]. Concerns about how to keep medical providers safe in the course of the COVID-19 pandemic might have been at least in part responsible for this decision.

Additionally, guidelines addressing the legal questions with regard to telemedicine in Austria are usually based on solutions from foreign legal systems that might not fit well into local regulations and/or be applicable only to one specialty medical field. Previously, the formulation of telemedicine guidelines based on local Austrian conditions and applicable to all medical fields was created in 2005 [17], but they have not yet been updated. Specific record-keeping and data security requirements are, however, still needed [18].

The consequences of the use of telemedicine on patients’ health-care outcomes and on medical practitioners are still not well understood, and there is little to no data available on the status in Austria specifically. Therefore, the research results at hand include data from GP practices to create an overarching understanding of the situation before and since the pandemic.

### Aims

Against this background, this paper aims to assess the use of VC, the up-to-dateness of practice websites and GPs’ views on whether eHealth is a positive aspect for the future of their practices in publicly funded primary health-care in Austria.

## Methods/Design

### Design

The data were collected as part of the PRICOV-19 study, a cross-sectional study using an online questionnaire sent to GPs in 37 European countries and Israel. In total, more than 4700 practices participated in this study. The PRICOV-19 study investigated how GP practices were organized during the COVID-19 pandemic to guarantee safe, effective, patient-centered, and equitable care. Also, the shift in roles and tasks and the wellbeing of staff members were researched [19].

The study was designed in accordance with the STROBE statement for cross-sectional studies [20] and was seen as safe by the ethics committee of the Medical University of Vienna (EC N°2200/2020).

### Recruitment

In Austria, the aim was to invite 500 GPs to participate. Therefore, we stratified all GPs with a contract with social health insurance companies according to the county they work in and their sex (similar to real distribution). Considering the GP and sex distribution within each county, we selected randomly 500 GPs via the platform Research Randomizer (www.randomizer.org) from a list provided by the Austrian Chamber of Physicians. However, with regards to Austrian data protection law this list did not contain any e-mail addresses, therefore we had to search for 500 email-addresses via official GP search engines (i.e. DocSearch.at etc.). Of the selected 500 GPs, only 300 had a publicly available e-mail address (others did not, or their addresses were incorrect). For this reason, we selected 400 GPs additionally with the same procedure and inclusion criteria as before and searched for their e-mail addresses in the list again. This way, we succeeded in collecting 500 e-mail addresses and invited these GPs accordingly to participate between December 2020 and July 2021. The invitation included a short description of the study and a link to the online survey, and four reminders were given. The Research Electronic Data Capture (REDCap) platform was used to host the questionnaire in all languages, to send out invitations to the national samples of GP practices, and to securely store the answers from the participants. A total 195 GPs began with the online questionnaire, and the relevant question for this analysis were completed by 176 (return rate 35.2%). This was higher than the median value of the response rate across all the PRICOV-countries (22.0%) [19].

### Questionnaire

The PRICOV-19 questionnaire was developed in multiple phases, including a pilot study in Belgium. The final version included 53 items divided into the following six sections: patient flow (including appointments, triage, and management for routine care), infection prevention, information processing, communication, collaboration and self-care, and practice and participant characteristics [19].

It was translated into the languages of the participating countries by the national coordinators. In Austria, the readability and feasibility of the translated questionnaire was checked by a group of GPs and colleagues (see acknowledgments), and the questionnaire was back-translated into English.

Most important for this analysis were the questions regarding the dependent variables, which were as follows:

- Video consultations: “To what extent did this practice use VC before the pandemic?” and “To what extent has this practice used VC since the pandemic?” The answer options were “never,” “less than once a week,” “weekly,” “daily,” and “multiple times a day.”
- Up-to-dateness of practice websites: “In the 12 months since the pandemic, how often has the information on the website of this practice been updated?” The answer options were “not at all,” “1 or 2 times,” “less than monthly,” “monthly,” “weekly,” “daily,” and “this practice does not have a website.”
- GPs’ views on whether eHealth is a positive aspect for the future of their practice: “What positive aspects for the future can you gain from the COVID-19 pandemic for your practice in terms of innovations in eHealth (e.g. video consultations)?” The answer options were “yes” and “no.”

Furthermore, the following background questions regarding the practice were relevant for the independent, exploratory variables:

- Work experience: “How many years of work experience do you have in general practice?”
- Number of GPs at the practice: “How many GPs and GP trainees are working at this practice? Count every GP and GP trainee as one, irrespective of whether they are full-time or not. Do not forget to include yourself.”
- Location of the practice: “How would you characterize the location of this practice?” The answer options were “big (inner)city,” “suburbs,” “(small) town,” “mixed urban–rural,” and “rural.”
- Size of the patient population: “We would like to get an idea of the size of this practice. How many patients are registered at this practice? If there are no registrations, please indicate the total practice population.”
- Patient population composition: “Compared to the average PC practice in your country, would you say that this practice on average treats more/fewer patients from the following categories: patients with a migration background with difficulty speaking the local language, patients with limited health literacy or low literacy, patients with financial problems, patients with a psychiatric vulnerability, patients over the age of 70, patients with chronic conditions?” The answer options were “above average,” “approximately the average,” and “below average.”

### Dependent variables

For this analysis, the answer options regarding the number of video consultations were clustered into “never or less than once per week” (category 0), “once per week” (category 1), and “daily or multiple times per day” (category 2) for both before and since the pandemic. For the binary regression model, only two categories were created, namely “never or less than once per week” (no video consultation) and “once per week, daily, or multiple times per day” (video consultation).

An additional variable was then built and named “increase in video consultations.” For this purpose, the given category for video consultations before the pandemic was subtracted from the given category for video consultations since the pandemic. Every positive result was counted as an increase.

Regarding up-to-datedness of the practice website, we clustered the answer options into no website, no up-to-date website (1 or 2 times, less than once per month), and up-to-date website (once per month, weekly, daily). For the binary regression model alone, two categories were built with “no website, 1 or 2 times, less than once per month” (not up-to-date) and “once per month, weekly, daily” (up-to-date). The views of GPs on whether eHeath is a positive aspect for the future of their practices stayed with the two answer categories, namely “yes” and “no.”

### Independent, exploratory variables

The work experience variable was clustered into three categories, namely 0 years to 4 years 11 months, 5 years to 14 years 11 months, and 15 years and more. The number of GPs working was also grouped into three categories, single-handed (1 GP), 2–3 GPs, and 4 or more GPs. The practice location was clustered into big cities, medium-sized locations (i.e., suburbs, small towns, and mixed), and rural areas. The size of the patient population was clustered into four groups: 0–1999 patients, 2000–4,999 patients, and 5000 patients or more. The patient population composition stayed with their answer options "above average,” “approximately the average,” and “below average.”

### Data analyses

First, all independent and dependent variables were described by descriptive statistical methods (frequencies and percentages). Subgroup analyses were then conducted by means of cross-table and Fisher’s exact tests because of the small numbers in several subgroups.

Binary logistic regression models were additionally used. In them, video consultations since the pandemic, up-to-date websites, and views on eHealth as the future were defined as dependent variables consecutively. In each regression model, all explanatory variables were taken into the model simultaneously. The results of all the regression models are presented as odds ratios with 95% confidence intervals. Nagelkerke’s R^2^ was presented as a measure of model fit.

### Ethical considerations

The study was seen as safe by the ethics committee of the Medical University of Vienna (EC N°2200/2020). All participants were classified as experts, and the survey was designed to be completely anonymous. All participants had to read the informed consent at the beginning of the questionnaire and could only fill out the questionnaire if they clicked the “I agree” button.

## Results

Table 1 shows that the overall use of VC was rare in Austria, both before and since the pandemic. In line with this, 93.9% of the facilities saw no increase in VC use.

**Table 1:** Description of the dependent variables

Variable	Subvariable	n	%
Video consultations before	never or less that once per week	128	96.2
	weekly	2	1.5
	daily or multiple times per day	3	2.3
Video consultations since	never or less than once per week	122	92.4
	weekly	5	3.8
	daily or multiple times per day	5	3.8
Increase video consultations	none	124	93.9
	one category more often	7	5.3
	two categories more often	1	0,8
Up-to-date practice webpage	no website	12	13.6
	no up-to-date website	37	42.0
	up-to-date website	39	44.3
Positive assumption for eHealth as future	yes	49	27.8
	no	127	72.2

Of the practices, 44.3% had an up-to-date webpage, and 27.8% assumed that the pandemic might have been a positive driver for eHealth in their practices.

Table 2 shows the characteristics of the participating GP practices. Half of the practices were located in rural areas, and 65.5% were run single-handedly. The patient population was no larger than 1999 in a little more than two-thirds of the practices. Of the participating GPs, 61.1% had work experience of more than 15 years. The GPs in nearly half of the practices estimated the number of patients with migrant backgrounds (who were not proficient in the German language) to be lower than the country average. Regarding the number of patients in all the other surveyed categories, GPs rated their own patient population as mainly among the average.

**Table 2:** Description of the independent, explanatory variables

Variable	Subvariable	n	%
Place of practice	big cities	30	21.4
	medium locations	40	28.6
	rural areas	70	50.0
N° GPs working in practice	single-handed (1 GP)	91	65.5
	2–3 GPs	35	25.2
	4 and more GPs	13	9.4
Size of patient population	0–1999	93	67.9
	2000–4999	33	24.1
	5000 +	11	8.0
GP years of experience	0y—4y11m	11	8.4
	5y—14y11m	40	30.5
	> 15y and more	80	61.1
Practices have patients with migration background	under country average	67	48.6
	average	45	32.6
	over average	26	18.8
…low health literacy	under country average	48	35.6
	average	74	54.8
	over average	13	9.6
…over 70 years of age	under country average	8	5.8
	average	91	65.5
	over average	40	28.8
…chronic diseases	under country average	0	0
	average	101	72.7
	over average	38	27.3
…psychiatric diseases	under country average	9	6.7
	average	104	77.0
	over average	22	16.3
… financial problems/poverty	under country average	25	18.7
	average	97	72.4
	over average	12	9.0

The results of the cross-table and Fisher’s exact tests showed that only a few significant associations could be reported (Table 3). Practices with fewer patients over the age of 70 than the average conducted significantly more VC than practices did on average. Additionally, practices with more than the average number of patients with chronic diseases reported using VC significantly more than practices did on average. For practices in big cities and those with higher numbers of GPs, compared with single-handed practices, a positive trend in VC could be seen.

**Table 3:** Associations between independent and explanatory variables

Variable	Subvariable	Video consultation since		Video consultation increase		Up-to-date webpage		eHealth as future	
		**Yes** (weekly**,** daily or multiple times per day **% (n)**	**No** (never or less that once per week) **% (n)**	**Yes** (one or two categories) **% (n)**	**None** **% (n)**	**Up-to-date webpage** **% (n)**	**No or no up-to-date webpage** **% (n)**	**Yes** **% (n)**	**No** **% (n)**
Place of practice	Big cities	17.9 (5)a	82.1 (23)a	14.3 (4)a	85.7 (24)a	50 (12)a	50 (12)	46.7 (14)a	53.3 (16)a
	Medium (suburbs small town, mixed)	5.4 (2)a,b	94.6 (35)a,b	5.4 (2)a,b	94.6 (35)a,b	40 (10)a	60 (15)	37.5 (15)a	62.5 (25)a
	rural	4.5 (3)b	95.5 (64)b	3.0 (2)b	97.0 (65)b	43.6 (39)a	56.4 (22)	28.6 (20)a	71.4 (50)a
p		.109		.106		.794		.197	
GPs working in the practice	single-handed	6.0 (5)a	94.0 (78)a	4.8 (4)a	95.2 (79)a	38.8 (19)a	61.2 (30)a	39.6 (36)a	60.4 (55)a
	medium 2–3	5.7 (2)a,b	94.3 (33)a,b	2.9 (1)a	97.1 (34)a	48 (12)a	52 (13)a	22.9 (8)a	77.1 (27)a
	lange 4 +	23.1 (3)b	76.9 (10)b	23.1 (3)b	76,9 (10)b	53.8 (7)a	46.2 (6)a	38.5 (5)a	61.5 (8)a
p		.126		.055		.548		.201	
Patients registered	0–1999	5.9 (5)a	94.1 (80)a	4.7 (4)a	95.3 (81)a	38.5 (20)a	61.5 (32)a	34.4 (32)a,b	65.6 (61) a,b
	2000–4999	9.1 (3)a	90.9 (30)a	6.1 (2)a	93.9 (31)a	44 (11)a	56 (14)a	30.3 (10)b	69.7 (23)b
	5000 and more	18.2 (11)a	81.8 (9)a	18.2 (2)a	81.8 (9)a	66.7 (6)a	33.3 (3)a	63.6 (7)a	36.4 (4)a
p		.253		.222		.306		.147	
GP years of experience	0a—4a11m	9.1 (1)a	90.9 (10)a	9.1 (1)a	90.9 (10)a	50 (3)a	50 (3)a	27.3 (3)a	72.7 (8)a
	5a—14a11m	2.7 (1)a	97.3 (36)a	0 a	100 (37)a	50 (16)a	50 (16)a	40 (16)a	60 (24)a
	> 15a and more	9.33 (7)a	90.7 (68)a	8 (6)a	92 (69)a	40 (18)a	60 (27)a	36.3 (29)a	63.7 (51)a
p		.403		.162		.653		.755	
Practices have patients with migration background	under country average	4.8 (3)a	95.2 (60)a	3.2 (2)a	96.8 (61)a	40.5 (15)a	59.5 (22)a	37.3 (25)a	62.7 (42)a
	average	9.5 (4)a	90.5 (38)a	7.1 (3)a	92.9 (39)a	41.9 (13)a	58.1 (18)a	31.1 (14)a	68.9 (31)a
	over average	12.0 (3)a	88.0 (22)a	12 (3)a	88 (22)a	57.9 (11)a	42.1 (8)a	38.5 (10)a	61.5 (16)a
p		.418		.205		.487		.794	
…low health literacy	under country average	4.5 (2)a	95.5 (42)a	4.5 (2)a	95.5 (42)a	38.2 (13)a	61.8 (21)a	29.2 (14)a	70.8 (34)a
	average	8.3 (6)a	91.7 (66)a	5.6 (4)a	94.4 (68)a	44.4 (20)a	55.6 (25)a	40.5 (30)a	59.5 (44)a
	over average	16.8 (2)a	83.3 (10)a	16.7 (2)a	83.3 (10)a	62.5 (5)a	37.5 (3)a	30.8 (4)a	69.2 (9)a
p		.307		.228		.481		.411	
…over 70	under country average	28.6 (2)a	71.4 (5)a	14.3 (1)a	85.7 (6)a	50 (2)a	50 (2)a	75 (6)a	25 (2)a
	average	3.5 (3)b	96.5 (82)b	3.5 (3)a	96.5 (82)a	41.7 (25)a	58.3 (35)a	34.1 (31)b	65.9 (60)b
	over average	12.8 (5)a,b	87.2 (34)a,b	10.3 (4)a	89.7 (35)a	47.8 (11)a	52.2 (12)a	30.0 (12)b	70 (28)b
p		.**021**		.190		.862		.055	
…chronic diseases	under country average	0	0	0	0	0	0	0	0
	average	4.3 (4)a	95.7 (90)a	3.2 (3)a	96.8 (91)a	41.5 (27)a	58.5 (38)a	40.6 (41)a	59.4 (60)a
	over average	16.2 (6)b	83.8 (31)b	13.5 (5)b	86.5 (32)b	50 (11)a	50 (11)a	21.1 (8)b	78.9 (30)b
p		**.030**		**.040**		.620		**.045**	
…psychiatric diseases	under country average	0 a	8 (100)a	0 a	100 (8)a	50 (3)a	50 (3)a	11.1 (1)a	88.9 (8)a
	average	7.1 (7)a	92.9 (91)a	6.1 (6)	93.9 (92)a	45.5 (30)a	54.5 (36)a	38.5 (40)a	61.5 (64)a
	over average	13.6 (3)a	86.4 (19)a	9.1 (2)a	90.9 (20)a	38.5 (5)a	61.5 (8)a	27.3 (6)a	72.7 (16)a
p		.452		.787		.926		.208	
… financial problems/poverty	under country average	0 a	21 (100)a	0 a	100 (21)a	21.1 (4)a	78.9 (15)a	28 (7)a	72 (18)a
	average	7.4 (7)a	92.6 (87)a	5.3 (5)a	94.7 (89)a	47.5 (28)b	52.5 (31)b	35.1 (34)a	64.9 (63)a
	over average	16.7 (2)a	83.3 (10)a	16.7 (2)a	83.3 (10)a	71.4 (5)b	28.6 (2)b	41.7 (5)a	58.3 (7)a
p		.133		.122		**.042**		.656	

a, b The letters following the percentages and total numbers (a, b) represent a subset of the variable category that is not significantly different at a significance level of *p* < 0.05 if it is the same subscript for the same variable

*p*-value significant at the level of *p* < 0.05

Regarding up-to-date websites, only possible financial problems of the patient population showed a difference. More specifically, practices with below-average related populations had an up-to-date website significantly less often than practices in or over the average.

Regarding the assumption that eHealth might be a positive aspect for the future, practices with a practice population with fewer patients over the age of 70 than average more often answered “yes” than practices on or above the average. In addition, contrary to VC, practices with an above average number of patients with chronic diseases viewed that eHealth was a positive aspect for the future significantly less often. A positive trend towards future eHealth can be seen for practices with 5000 patients and more.

Table 4 shows compared to Table 3 that having a population size of 5000 patients and more results in the binary regression model with all explanatory variables concomitantly included in a significantly higher probability of seeing eHealth as the future for the practice.

**Table 4:** Results of the binary logistic regression models

Variable	Sub-variable	Video consultations since OR [95% CI], p	Up-to-date webpage OR [95% CI], p	eHealth as future OR [95% CI], p
Place of practice	big cities	1.0	1.0	1.0
	medium locations	2.4 [0.1,58.3], *p* = 0.59	0.4 [0.1, 2.3], *p* = 0.44	0.8 [0.2,3.1], *p* = 0.76
	rural area	3.3 [0.1,111.3], *p* = 0.50	0.5 [0.1, 2.7], *p* = 0.43	0.5 [0.1,2.0], *p* = 0.32
N° GPs working in the practice	single-handed	1.0	1.0	1.0
	2–3 GPs	1.6 [0.1,34.4], *p* = 0.75	1.8 [0.5, 6.7], *p* = 0.39	0.4 [0.1,1.3], *p* = 0.14
	4 and more GPs	13.7 [0.1,1651.0], *p* = 0.29	2.1 [0.3, 14.5], *p* = 0.45	1.6 [0.3,10.3], *p* = 0.60
Size of patient population	0–1999	1.0	1.0	1.0
	2000–4999	0.20 [0.0, 5.4], *p* = 0.34	0.8 [0.2, 3.1], *p* = 0.74	0.6 [0.2, 2.0], *p* = 0.39
	5000 +	27.1 [0.6, 1330.3], *p* = 0.10	2.4 [0.4, 16.7], *p* = 0.37	6.9 [1.0, 46.7], ***p* < 0.05**
GP years of experience	0a—4a11m	0.4 [0.0,13.6], *p* = 0.60	2.6 [0.3, 23.5], *p* = 0.40	0.3 [0.0, 1.7], *p* = 0.16
	5a—14a11m	0.4 [0.0,19.8], *p* = 0.61	2.8 [0.7, 10.8], *p* = 0.13	1.2 [0.4, 3.5], *p* = .69
	> 15a and more	1.0	1.0	1.0
Practices have patients with migration background	under country average	1.0	1.0	1.0
	average	0.4 [0.0, 13.1], *p* = 0.60	0.9 [0.2, 4.8]. *p* = 0.87	0.4 [0.1,1.4], *p* = 0.15
	over average	4.9 [0.1, 196.9], *p* = 0.40	3.4 [0.5, 23.3], *p* = 0.21	0.7 [0.2,3.1], *p* = .60
…low health literacy	under country average	1.0	1.0	1.0
	average	46.0 [0.1,16890.0], *p* = 0.20	0.5 [0.1, 2.5], *p* = 0.40	2.3 [0.7, 7.7], *p* = 0.18
	over average	130.5 [0.1, 256220.5], *p* = 0.21	0.2 [0.0, 7.0], *p* = 0.42	0.5 [0.0, 9.3], *p* = .67
…over 70 years of age	under country average	1.0	1.0	1.0
	average	0.0 [0.0, 0.221], ***p* = 0.02**	0.4 [0.0, 4.8], *p* = 0.44	0.1 [0.0,1.3], *p* = .08
	over average	0.0 [0.0, 0.2], ***p* = 0.02**	0.4 [0.0, 8.1], *p* = 0.52	0.2 [0.0, 3.3], *p* = 0.28
…chronic disease	average	1.0	1.0	1.0
	over average	597.2 [2.1, 173682.4], ***p* = 0.03**	2.9 [0.4, 21.4], *p* = 0.31	0.2 [0.1, 1.1], *p* = 0.07
…psychiatric diseases	under country average	1.0	1.0	1.0
	average	na, *p* = 1.0	0.3 [0.0, 3.9], *p* = 0.33	4.8 [0.4, 54.0], *p* = .20
	over average	na, *p* = 1.0	0.1 [0.0, 1.5], *p* = 0.09	3.5 [0.2, 51.6], *p* = 0.36
… financial problems/poverty	under country average	1.0	1.0	1.0
	average	na, *p* = 1.0	7.9 [1.0, 61.3], ***p* < 0.05**	0.9 [0.2, 3.7], *p* = 0.90
	over average	na, *p* = 1.0	49.8 [0.5, 5025.8], *p* = 0.10	1.6 [0.1, 35.7], *p* = 0.76
Nagelkerkes R^2^		0.519	0.313	0.324

*P-value* significant at the level of *p* < 0.05

## Discussion

Altogether, the use of VC was observed to be extremely low in Austria. Even since the pandemic, which required safer forms of consultation, the use of VC has remained very low and has not increased substantially across all the groups analyzed (Tables 1 and 3). In addition, during the recruitment procedure, we observed that only about two-thirds of the GPs we wanted to invite had a publicly available or up-to-date e-mail address; of these, less than half had an up-to-date webpage, and 13.6% did not even have a website (Table 1). Reason for adding “up-to-date website” as a variable was the assumption that GPs, as health advocates, who keep an eye on a state-of-the-art online presence tend to have higher IT affinity than GPs who don’t [21]. Hence, when it comes to the assumption that eHealth might be a positive aspect for the future of their practices, less than one-third of the participants agreed (Table 1).

This leads to speculation that the general preparedness and readiness for VC and eHealth among Austrian general practices is not yet certain. Greenhalgh et al. stated in 2020 [10, 11, 22] that readiness for telemedicine and digitalization can only be as good as the surrounding environment that has prepared for it. In Austria, the infrastructure for internet services such as high-speed internet access and computers with web cameras is largely in place. However, implementing proper eHealth environments, including VC, has not been a national priority for the healthcare system. In particular, when it comes to VC, several country-level pre-conditions have to be guaranteed, namely adequate guidelines, frameworks for data protection security, and transparent pathways regarding accountability in cases of adverse events. At the practice level, the availability of user-friendly software programs and the ability to easily embed the VC software into the patient’s software are of great importance. Additionally, training should be made available for physicians and patients. Finally, adequate payment models that should also respect the additional organizational time needed before VC should be enabled [10, 11, 22–24]. Presently, none of this has been comprehensively implemented in Austria, neither for VCs nor for other telemedicine options, such as telemonitoring, health app use, and the implementation of shareable electronic health records. However, the need and willingness are there, and the first pilot projects, such as that of Herz Tirol, have shown promising results [25–27]. A payment position for teleconsultations at the same level as face-to-face consultations was introduced only in late 2020, and the first software program for VC, created by the Main Association of the Social Health Insurance Companies, is at the testing phase [25, 28].

Moreover, a major issue in this regard is the complicated funding structure of VC pilot projects in Austria. There is an unavoidable funding barrier between the private and public sectors that does not allow the instant implementation of promising solutions from start-ups to be used in the insurance-funded public health-care system.

On the other hand, there also might still be skepticism and fear of change on the physicians’ side regarding digitalization processes. Evaluating the acceptance of the need for electronic health-care records, for example, was the main goal of a study conducted by Hackl et al. [29]. The author hypothesized that negative preconceptions about electronic health-care records could create a fear of change, which might be exacerbated by a lack of information.

Also, from the patient’s side, an Austrian study showed that patients mainly used the telephone instead of video calling when contacting their doctors during the pandemic [30].

Despite the overall low use of video consultations, we could find a significant difference among practices with a below-average population of elderly patients over the age of 70 and those with an above-average number of patients with chronic diseases, in that they used VC more often. Practices with fewer patients over the age of 70 than the average also tended to see eHealth as a positive aspect for the future of their practice (Tables 3 and 4). These insights from the GPs side are in opposition to the views of older patients assessed in a study conducted by Bhatia et al. [31] of 65- to 75-year-olds in the greater metropolitan Boston area. These participants wanted VC to remain an option for primary health care, since transportation challenges increase for face-to-face appointments with their GPs. One major benefit was the opportunity to include family members in a VC. The willingness of patients to use VC did not seem to be dependent on the patient’s age or gender but rather on their personality characteristics with respect to health information and their desire to stay in touch with their physician, which implies that a certain amount of success lies in eHealth literacy competence and proper support by the environment for this [4, 32].

A paradoxical finding in this study was that while practices with an above average number of patients with chronic diseases used VC significantly more often, they less frequently saw eHealth as a positive future aspect for their practices than practices did on average. It could be speculated that this might relate to how VCs are conducted and because they are time consuming, in particular with patients with complex and/or chronic diseases. Therefore, these patients might not be the best suited for VC in the Austrian healthcare system with its established incentives.

Additionally, we could see a trend for bigger facilities with more GPs in bigger cities using VC more often and also viewing eHealth more often as the future for their practices.

### Strengths and limitations

The main strength of the paper is that it is the first of its kind in Austria. This study was part of an international project and succeeded in recruiting a random sample in Austria. However, the rather low return rate could imply that only highly motivated GPs participated. This means that our results might be an overestimation. On the other hand, it also could be that GPs who were massively involved with the pandemic and had no time available to answer the questionnaire were not represented. This might lead to speculation that particularly motivated GPs did not answer the questionnaire, which could have led to an underestimation of our results.

Moreover, our recruitment procedure led to the exclusion of GPs without a working e-mail address. Assuming that these GPs are generally uninterested in digitization processes, this could again lead to the assumption that our findings are overestimations. Additionally, recall bias is a general problem in surveys [33].

Additional qualitative research is urgently needed to get a deeper insight into the major causes for our findings.

## Conclusions

The use of VC in general practice and readiness for other telemedicine approaches is very low in Austria. Austria has to urgently take the example of countries with a transparent and comprehensive national digital health strategy, including VC. Without proper payment, patient and physician inclusion, and support with regard to administrative and organizational aspects, no substantial change will occur in spite of there being an increase in need due to the pandemic and changes in the patient population.

VC and other eHealth technologies will be a key resource in patient treatment in the future, but without proper research and implementation strategies, it will be a usage lottery for patients and general practitioners alike. The lack of an international implementation framework adapted to Austria’s unique funding system and legal requirements will be a major barrier to adequate knowledge transfer. The question is not whether VC will find its way to GP practices or primary healthcare facilities but rather when and how.

## Data Availability

All data are centrally stored on the server of Ghent University (Belgium). All data was anonymized at Ghent University, and all raw data that could lead to the identification of the respondents was permanently removed. Reasonable request is required to access non-identifiable data by users who are external to the PRICOV-19 consortium. Access will be subject to a data transfer agreement and following approval from the principal investigator of the PRICOV-19 study.

## Abbreviations

COVID-19: Corona virus diseases 2019
eHealth: The use of information and communication technology to support health and healthcare
GP: General Practitioner
IT: Information technology
PRICOV19 study: The name for a cross-sectional study in 38 countries on the organization of care in general practices during the COVID-19 pandemic
REDCap platform: Research Electronic Data Capture platform
SARS: Severe acute respiratory syndrome
SARS-CoV2: Severe acute respiratory syndrome coronavirus 2
VC: Video consultation
